# Challenges in realizing ultraflat materials surfaces

**DOI:** 10.3762/bjnano.4.99

**Published:** 2013-12-11

**Authors:** Takashi Yatsui, Wataru Nomura, Fabrice Stehlin, Olivier Soppera, Makoto Naruse, Motoichi Ohtsu

**Affiliations:** 1 School of Engineering, University of Tokyo, Bunkyo-ku, Tokyo 113-8656, Japan; 2 Advanced Low Carbon Technology Research and Development Program, Japan Science and Technology Agency, 7, Gobancho, Chiyoda-ku, Tokyo 102-0076, Japan; 3 Institut de Sciences des Materiaux de Mulhouse (IS2M), CNRS UMR 7361, 15, rue Jean Starcky, BP 2488, Mulhouse Cedex 68057, France; 4 National Institute of Information and Communications Technology, 4-2-1 Nukui-kita, Koganei, Tokyo 184-8795, Japan

**Keywords:** dressed photon–phonon, phonon-assisted process, polishing, self-organized process

## Abstract

Ultraflat surface substrates are required to achieve an optimal performance of future optical, electronic, or optoelectronic devices for various applications, because such surfaces reduce the scattering loss of photons, electrons, or both at the surfaces and interfaces. In this paper, we review recent progress toward the realization of ultraflat materials surfaces. First, we review the development of surface-flattening techniques. Second, we briefly review the dressed photon–phonon (DPP), a nanometric quasiparticle that describes the coupled state of a photon, an electron, and a multimode-coherent phonon. Then, we review several recent developments based on DPP-photochemical etching and desorption processes, which have resulted in angstrom-scale flat surfaces. To confirm that the superior flatness of these surfaces that originated from the DPP process, we also review a simplified mathematical model that describes the scale-dependent effects of optical near-fields. Finally, we present the future outlook for these technologies.

## Review

### Introduction

In order to improve device performance and to conserve energy, a reduction of the surface roughness (*R*_a_) is the most important challenge for the future of the electronic and opto-electronic industry. As for the optics in the extreme ultraviolet (EUV) region, in which the wavelength is extremely short, i.e., down to values of 13.4 nm, *R*_a_ must be brought down to around 1 Å in order to reduce the light-scattering loss [1]. The use of ultraflat mirrors is expected to help in realizing the high-power lasers that are required for future applications such as EUV system [2]. In addition, the necessity of shortening the pulse widths of lasers is a major topic in the field of laser-machining processes, in which a flattened mirror can increase the laser durability [3]. The electron scattering losses must also be reduced for various industrial and scientific applications. To realize high-power light-emitting diodes (LEDs), the surface roughness of the substrate can be a serious problem, because substrates with large *R*_a_ values induce defects or dislocations in the deposited active layer [4]. Diamond is a promising material for future power devices because of its many excellent characteristics including high values for hardness and thermal conductivity, and excellent semiconductor properties such as a high dielectric breakdown field and a high carrier mobility [5]. However, at the same time, the hardness of diamond makes it difficult to realize a flattened surface, and therefore the performance of diamond devices has not been as good as expected. Furthermore, diamond is also a promising material for future quantum computing, because diamond with nitrogen vacancies can be a stable single-photon emitter at room temperature [6]. However, the high surface roughness of the diamond due to its hardness limits its performance.

Conventionally, mechanical polishing has been used to flatten surfaces. However, this method is generally limited to reducing *R*_a_ to around several angstroms, because the minimum value is governed by the roughness of the polishing pad, which is on the order of 10 μm, or the diameter of the polishing particles in the slurry, which can be as small as 100 nm. The slurries that are used for chemical–mechanical polishing (CMP) [7] consist of a large amount of the rare-earth material CeO_2_, which chemically polishes the substrate. Owing to issues of cost and material availability, there has recently been an effort to reduce the usage of such rare-earth materials [8]. To reduce the usage of the CeO_2_, many groups have attempted to develop alternative polishing pads [9] and slurries [10]. Watanabe et al. developed a surface treatment for SiC and diamond that uses a photocatalytic effect [11]. To induce this photocatalytic effect, they use a light source of shorter wavelengths to excite the carriers in TiO_2_, so that the generated electrons and holes induce a photocatalytic effect and etch the substrate [12]. Those techniques resulted in ultraflat surfaces with *R*_a_ values as small as 2 Å. Although CeO_2_ is not required in this technique, it does require a polishing pad to heat the substrate through friction between the polishing pad and substrate. Thus, the light must be introduced through the substrate, which should therefore be thin. Furthermore, mechanical polishing causes surface damage (scratches or pits) when the polishing particles and/or impurities in the slurry abrade the substrate.

A recent increase in electron mobility was achieved by introducing Ge in an Si device [13]. The higher electron mobility was realized through the modification of the band structure by in-plane tensile strain due to the wider interatomic distance for Ge compared to Si [14]. Furthermore, the wider interatomic distance induced a stretching force and resulted in a flattening of the interface between Si and Ge. However, the surface still remained rough. These problems can be overcome by eliminating contact polishing entirely. One promising approach for reducing the surface roughness is ion-beam smoothing [15]. Ion-beam irradiation at angles that are near grazing incidence preferentially removes large protrusions from the surface. This way a smoothing of wide areas can be achieved, while the surface damage is reduced. In addition, the use of a clustered ion beam to reduce the surface damage can lead to ultraflat surfaces of several hundred mm in diameter with a small *R*_a_ of 1 Å [16]. Although ion-beam smoothing does not require a polishing pad, it can still cause damage due to ion bombardment, and this technique also requires high-vacuum conditions, which is another obstacle to its widespread application.

In the context of these challenges, researchers have developed nanophotonic methods as alternative polishing techniques. Before reviewing recent studies of nanophotonic smoothing, we first provide an overview of the development of nanophotonics in the next section.

### Optical near field: dressed photon–phonon

Near-field optics has made it possible to reduce the size of photonic devices to the sub-wavelength scale or smaller [17]. In particular, nanoscale photonic devices such as AND-gates, NOT-gates, and focusing devices have been developed that utilize the optical near field generated in nanoscale semiconductor quantum structures and the dipole-forbidden near-field energy transfer. Moreover, near-field optics has been used to fabricate nanoscale structures beyond the diffraction limit of light. For example, photolithography has been used to fabricate structures of several tens of nanometres in size by introducing near fields with the use of a visible light source [18]. Such advances can lead to the realization of systems that do not require EUV light sources, which are currently of limited practical use in industry because the equipment involved is large and expensive [19]. Thus, with further development, near-field lithography will be able to satisfy the requirements of future semiconductor electronic devices, such as highly integrated DRAMs.

**Figure 1:**
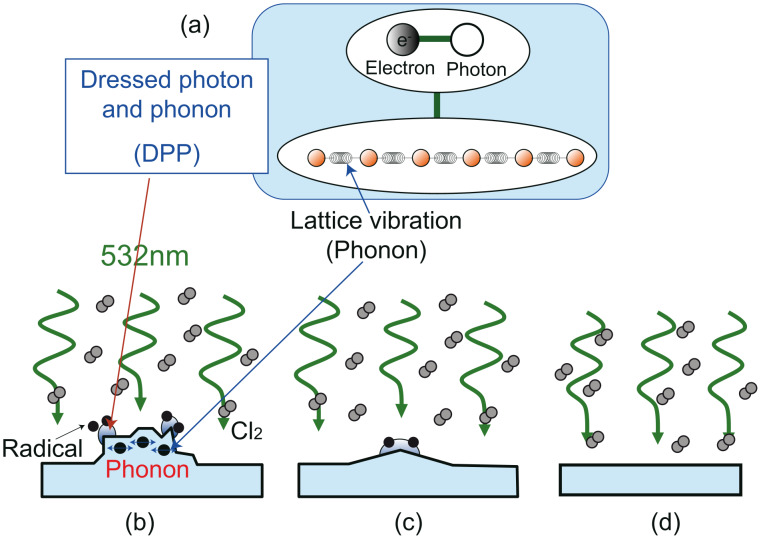
(a) Schematic diagram of a dressed photon–phonon (DPP). (b–d) Schematic diagrams of DPP etching. The etching gas is selectively photodissociated at the protrusions. The activated etching gas atoms etch away the protrusions. DPP etching stops automatically when the substrate becomes sufficiently flat.

The physics of these nanoscale optics has been developed under the assumption of a conventional multipolar quantum electrodynamic Hamiltonian in a Coulomb gauge and of single-particle states in a finite nanosystem [20]. In such a system, fluctuations in the electromagnetic field (e.g., zero-point fluctuations of the vacuum) cause nanomaterials to emit or absorb virtual photons, i.e., the optical near fields are continuously present around illuminated materials. These so-called virtual absorption and emission processes violate the energy conservation law but are consistent with the Heisenberg uncertainty principle, and to take these processes into account, nanomaterial can be considered to be covered with a cloud of virtual photons. Within this framework, the virtual photon can be described as a coupled state of an electron and a real photon (i.e., a free photon (FP); Figure 1a). This virtual photon, also referred to as a *dressed photon* (DP) [21], is distinguished from the FP because it carries a material excitation energy. Therefore, the energy of the DP, *h*ν_DP_, is larger than that of the FP, *h*ν_DP_.

To take advantage of nanoscale optics, a thorough understanding of the nanoscale material is required. Such nanoscale materials are composed of a crystal lattice, and after a DP is generated at the surface of an illuminated nanoscale particle, its energy can be exchanged with this crystal lattice. Through this exchange, vibrational modes can be coherently excited in the crystal lattice, creating multiple modes of coherent phonon states [22]. Consequently, the DP and a coherent phonon form a coupled state (Figure 1a). This state (the dressed photon and a phonon: DPP) constitutes a quasi-particle that is generated only when the particle size is sufficiently small so that the crystal lattice vibration is excited coherently. In contrast, vibrational modes cannot be excited coherently in bulk materials, and energy is instead dissipated as heat throughout the material. Therefore, the energy of the DPP, *h*ν_DPP_, is higher than *h*ν_DP_ (*h*ν_FP_ < *h*ν_DP_ < *h*ν_DPP_).

This DPP theory has been used to explain numerous experiments on topics such as photochemical vapour deposition [23], photolithography [24], and visible-light water splitting [25], as well as studies on photovoltaic devices [26] and energy up-conversion devices [27]. The efficiency of energy up-conversion by using DPP is reported to be more than three-fold higher than that of up-conversion by using conventional two-photon absorption for the generation of second harmonics, because the phonon state cannot be coupled with propagating light in the far-field [27]. Furthermore, DPPs have been reported to be localized selectively at disordered nanostructures such as impurity sites or protrusion edges [28].

As described above, the principles and concepts of DPP technology differ significantly from those of conventional wave-optical technologies such as photonic crystals [29], plasmonics [30], metamaterials [31], or quantum-dot photonic devices [32], in which the size and function are governed by the light diffraction limit. Therefore, we next use the framework of the DPP theory to review a nanophotonic fabrication process that realizes angstrom-scale flattening of substrate surfaces.

### Dressed photon–phonon etching

DPPs can be consistently generated by irradiating a rough material surface with nanoscale structures. The generated DPPs induce the photodissociation of molecules at protrusions on the substrate (Figure 1b) even when the incident photon energy is smaller than the photodissociation energy, *E*_d_. The dissociated molecules in turn induce the etching of the protrusion and the flattening the substrate (Figure 1c). This etching process stops automatically when the surface becomes flat and more homogeneous, because then the DPPs disappear. Therefore, surface smoothing by utilising DPPs is a self-organized process [26].

**Figure 2:**
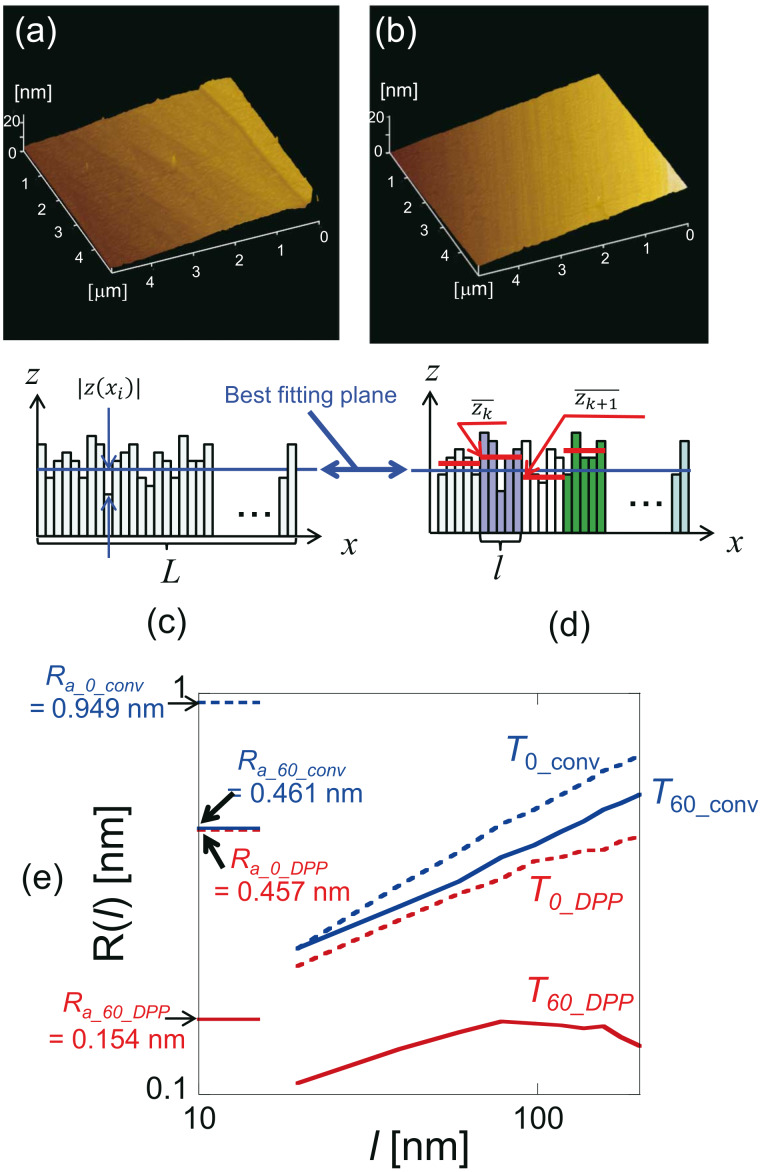
Typical atomic force microscopy (AFM) images of a type-Ib diamond (111) substrate with a 5 μm × 5 μm scanning area. The images were 256 × 256 pixels, which corresponds to a spatial resolution of 20 nm. AFM images were obtained for etching times of (a) 0 min (before etching) and (b) 60 min (after etching). The corresponding surface roughness values *R*_a_ were 0.457 nm and 0.154 nm, respectively. Schematic diagrams of the (c) surface roughness *R_a_* and (d) standard deviation *R*(*l*). (e) Calculated values of *R*(*l*). Dashed and solid red lines show values before (*T*_0_DPP_ (0 min)) and after (*T*_60_DPP_ (60 min)) DPP etching (3.81 eV), respectively. Dashed and solid blue lines show values before (*T*_0_conv_ (0 min)) and after (*T*_60_ conv_ (60 min)) conventional adiabatic etching (5.82 eV), respectively. Reproduced with permission from [33]. Copyright 2012 IOP Publishing.

The DPP etching technique was developed to smooth various materials, including SiO_2_ (fused silica and soda lime glass), plastic films, and crystal substrates. DPP etching on a diamond substrate [33] was performed by using O_2_ gas, which has an *E*_d_ of 5.12 eV (wavelength, λ, of 242 nm) [34]. A continuous-wave (CW) He–Cd laser (λ = 325 nm, 3.81 eV, excitation power 0.8 W/cm^2^) was used to dissociate the O_2_ gas in the DPP etching, which produced the oxygen radicals O* to etch the protrusions of the diamond substrate and ultimately yielded an ultra-flat surface. Since the photon energy of the laser is lower than *E*_d_ of O_2_, the conventional O_2_ adiabatic photochemical reaction was avoided. Furthermore, the laser power density of approx. 1 W·cm^−2^ was 10^15^ times smaller than that associated with multi-photon processes using ultra-short pulse (femtosecond) lasers [35]. Therefore, the DPP etching process cannot be attributed to conventional multi-photon excitation processes [36]. A comparison of atomic force microscopy (AFM) images before (Figure 2a) and after 60 min of DPP etching (Figure 2b) confirmed that this treatment resulted in an ultra-flat surface with a small *R*_a_ value of 0.154 nm. The minimum value of *R*_a_ is to be determined by the interatomic distance. As reported in [33], the surface roughness after 30 min was 0.181 nm, which is almost as small as that after 60 min (0.154 nm). These values are comparable to the interatomic distance of 0.206 nm for (111) diamond [37], which indicates that the surface roughness reduction might be completed already after 30 min of etching. This was also supported by the fact that the *R*_a_ value remained the same after 24 hours of etching.

To verify that the smoothing effect originated from the DPP process, the surface roughness was compared by using AFM images taken after conventional photoetching, in which a photon energy higher than *E*_d_ was used and after DPP etching (i.e., nonadiabatic photoetching). We note that in the AFM images shown, the tilt in the scan was compensated by using the third-order least-squares method. The light source for the conventional photoetching was a 5.82 eV light (λ = 213 nm; 20 Hz; pulse width 5 ns), the energy of which was higher than *E*_d_ of O_2_ (5.12 eV). This light source induced adiabatic photodissociation of the O_2_ molecules. For this comparison, instead of using the usual value of *R_a_*, which is the average value of the absolute surface height deviations from the best-fitting plane (dashed blue line in Figure 2c), we developed a simplified mathematical model to describe the scale-dependent DPP effect. The value of *R_a_* is determined as


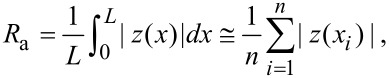


where |*z*(*x_i_*)| are the absolute deviations from the best-fitting plane, *L* is the evaluation length, *dx* is the spatial resolution of *z*(*x*), and *n* (= s*L*/*dx*) is the number of pixels in the measurement. *R_a_* thus provides information about the average surface roughness for the entire scanning region. The standard deviation of the height difference function is given by


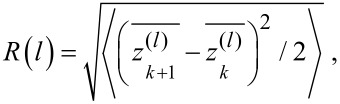


where *l* is the bin size, *z* is the height from the best-fitting plane, and 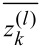 is the average *z* value of the bin (Figure 2d) [38]. This *R*(*l*) can be used to determine the contributions of the surface roughness values at different length scales to the overall surface roughness.

The red curves labelled *T*_0_DPP_ and *T*_60_DPP_ in Figure 2e show the calculated *R*(*l*) after 0 and 60 min of DPP etching, respectively. These results show that *R*(*l*) decreased for all scales of *l*. Furthermore, the *R*(*l*) values were comparable in magnitude to the *R*_a_ values shown on the left side in Figure 2e and they decreased as *R*_a_ decreased. The blue curves labelled *T*_0_conv_ and *T*_60_conv_ in Figure 2e represent the *R*(*l*) values calculated after 0 and 60 min of conventional photochemical etching, respectively. Etching for 60 min with 5.82 eV light (conventional adiabatic photochemical etching) resulted in a marked reduction in surface roughness from 0.949 nm (*R*_a_0_conv_) to 0.461 nm (*R*_a_60_conv_), as shown on the left side of Figure 2e. However, comparing the *R*(*l*) curves for 5.82 eV etching (*T*_0_conv_ (0 min) and *T*_60_conv_ (60 min)) revealed that *R*(*l*) was unchanged at *l* = 20 nm. Since the apex of the protrusion has a larger surface area and thus a higher etching rate, a reduction in the surface roughness is expected. However, the 5.82 eV light induced an adiabatic photochemical reaction in the gas-phase molecules, and there was no selective etching in this case. Therefore, etching with the 5.82 eV light did not the change the small-scale surface roughness profile. Moreover, it is noteworthy that this information could be revealed only by considering the *R*(*l*) values. The results shown in Figure 2e also indicate that conventional photochemical etching changes the large-scale surface roughness profile. Therefore, if the initial structure has a large surface roughness, at the beginning states, conventional photochemical etching can reduce the large-scale surface roughness faster than DPP etching only.

**Figure 3:**
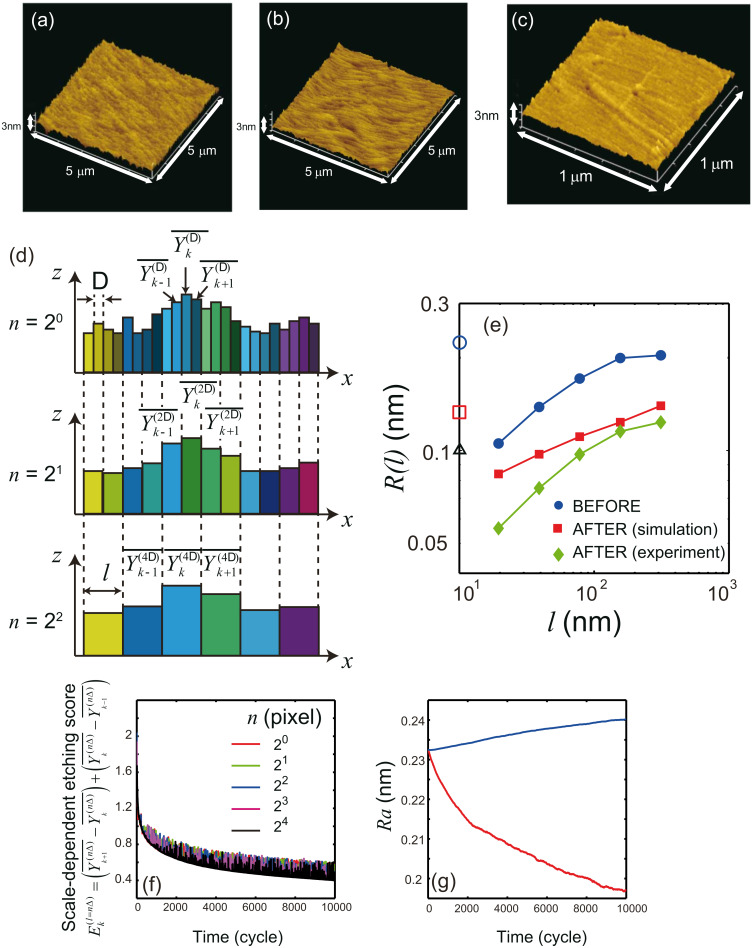
AFM images of the GaN surface (a) before and (b) after DPP etching. (c) Enlarged view (1.0 μm × 1.0 μm area) of the etched GaN surface. (d) Evaluation of surface roughness considering scale dependence. (e) Calculated *R*(*l*). Solid blue circles denote results before DPP etching; solid green diamonds, after DPP etching; and solid red squares, after 10,000 cycles of virtual etching. (f) Time evolution of the scale-dependent etching score 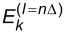 (see main text). (g) Calculated time evolution of *R*_a_ during virtual etching using the scale-dependent etching score. Red denotes the results for the multidimensional scale considering all etching scores, 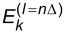
(*n* = 2*^m^*, *m* = 0 to 4), and blue, the unidimensional scale considering only finest-scale etching score 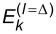.

DPP etching was also performed on GaN(001) substrates while using Cl_2_ gas at a pressure of 200 Pa. A 532 nm light (2.33 eV, CW laser, power density of 0.28 W·cm^−2^) was used for this photochemical etching, because *E*_d_ of Cl_2_ is 3.10 eV (which corresponds to a wavelength of 400 nm) [39]. The low power density also prevented any multiphoton excitation associated with irradiation from ultrashort-pulse lasers. The AFM images taken before (Figure 3a) and after 30 min (Figure 3b) of etching show that *R*_a_ decreased from 0.23 to 0.14 nm. GaN is a compound semiconductor, so the *R*_a_ value of 0.140 nm obtained for GaN might be limited by the value of the interatomic distance between Ga and N of 0.195 nm for hexagonal GaN [40]. Furthermore, *R*(*l*) again shows the individual contributions of the surface roughness at different length scales to the overall surface roughness, as shown in Figure 3e. In this figure, the solid blue circles and solid green diamonds in represent the *R*(*l*) values before the etching began (corresponding to the AFM image in Figure 3a) and after 30 min into the etching process (corresponding to the AFM image in Figure 3b), respectively. The horizontal axis corresponds to the scale *l* in units of length. It can be seen that *R*(*l*) decreased at both finer and broader scales. In addition, the open blue circle and open red square in Figure 3e correspond to the *R*_a_ values for Figure 3a and Figure 3b, respectively. The *R*(*l*) values were comparable to the *R*_a_ values and decreased as *R*_a_ decreased. It is noteworthy that *R*(*l*) decreased to less than 0.10 nm, which indicates that an ultrasmooth surface was obtained. This result is supported by the fact that *R*_a_ also decreased to 0.10 nm (open black triangle in Figure 3e for the smaller (1.0 μm × 1.0 μm) scanned area in Figure 3c).

To simulate the time evolution of the surface roughness and evaluate the scale-dependent attributes of DPP etching, we developed a *scale-dependent etching score* defined as


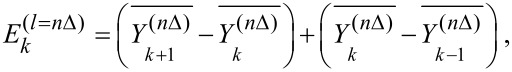


where Δ is the spatial resolution of the AFM image and the scale *l* is defined as *n*Δ, *Y* is the height, and 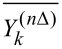 is the average *Y* value of the pixels, and *n* is the number of pixels (Figure 3d). In this approach, a highly convex region exhibits a larger score than regions that are more flat, which is useful because etching is more likely to occur at these sites. By using this scoring system, a virtual etching process was repeated and the etched surface profile was compared with the initial surface profile in the same experiment (Figure 3a). Meanwhile, a multiscale etching score was evolved for scales of *n* = 2*^m^* pixels (*m* = 0 to 4), as shown in Figure 3f. Over 10,000 repetition cycles, the surface height profile produced the *R*(*l*) values shown by the solid red squares in Figure 3e, which are consistent with the experimental results. Furthermore, the time evolution of *R*_a_ was investigated by calculating the scale-dependent etching scores (Figure 3f). The calculated *R*_a_ values decrease with the etching time when all etching scores 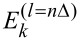 (*n* = 2*^m^*, *m* = 0 to 4) are considered (red curve in Figure 3g). In comparison, when the progress of the etching depended only on the etching score at the finest scale (*n* = 2^0^), the calculated *R*_a_ values (considering the finest etching score 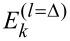) increase (blue curve in Figure 3g), whereas the finest-scale etching score decreased over time. This is another clear manifestation of the scale-dependent nature of optical near-fields and of the crucial role they play in DPP etching.

**Figure 4:**
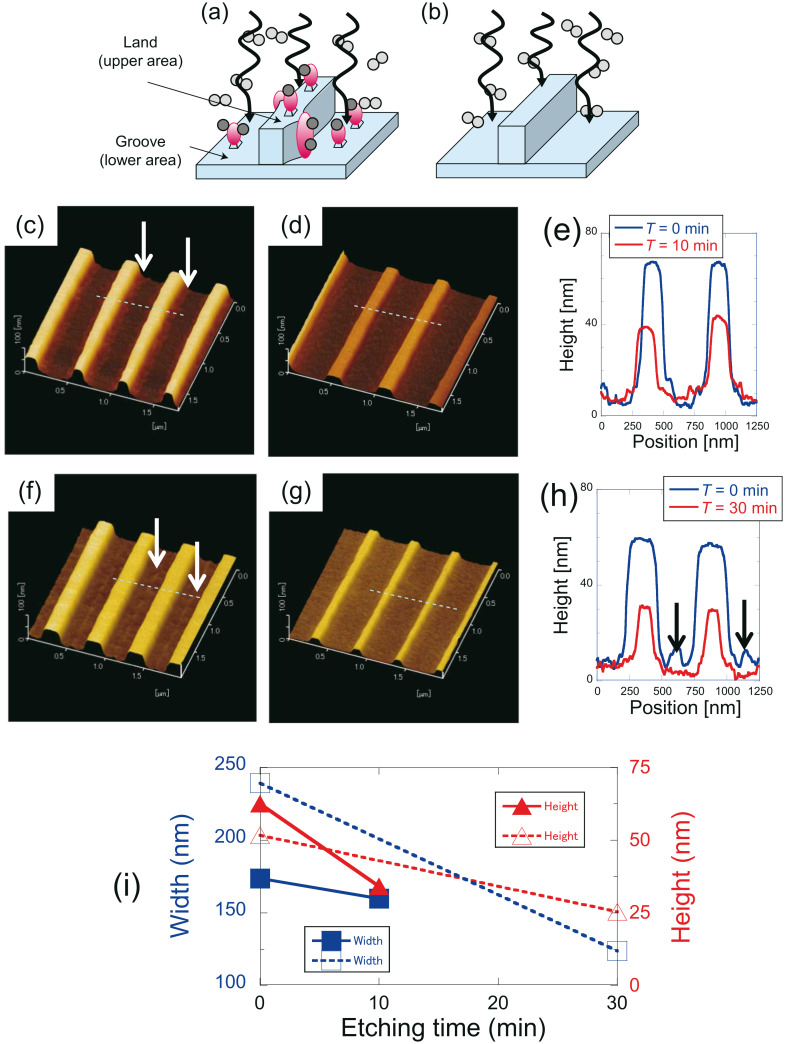
(a, b) Schematic diagrams of the DPP etching of substrates with nanostripe patterns. AFM images of TiO_2_ (c, f) before and (d, g) after DPP etching (after etching times of (d) 10 min and (g) 30 min). (e) Cross-sectional profiles along the white dashed lines in (c) and (d). (h) Cross-sectional profiles along the white dashed lines in (f) and (g). (i) Dependence of corrugation width and height on the etching time. Blue solid squares and red solid triangles are data for (c) and (d), blue open squares and red open triangles are for (f) and (g).

DPP etching is potentially applicable to various three-dimensional surfaces including concave and convex lenses, diffraction gratings, and the inner wall surfaces of cylinders, because it is a non-contact method, i.e., it does not require polishing pads. These potential applications have been confirmed by using the procedure to smooth a substrate with a nanostripe corrugation pattern (Figure 4a and Figure 4b). In particular, the side walls of diffraction grating corrugations in soda lime glass were polished by using DPP etching [41]. Consequently, the *R*_a_ values decreased for both the substrate and the grooved surface, and an additional reduction in the line edge roughness was observed. Another application of this technique involved the fabrication of a nanostripe pattern on TiO_2_. Direct ArF-laser photopatterning was followed by the application of a sol–gel negative tone photoresist to produce TiO_2_ nanostructures by using deep-UV (DUV) direct-write imaging [42–43]. Figure 4c and Figure 4f show representative AFM images taken at different positions (positions A and B, respectively) of a TiO_2_ sol–gel photoresist nanostripe corrugation pattern on a Si wafer.

DPP etching was performed under CW laser illumination (λ = 532 nm; power density (spatially uniform) 0.28 W·cm^−2^) and Cl_2_ gas exposure. Figure 4d and Figure 4f show typical AFM images after 10 min and 30 min of DPP etching, where reductions in both the width and the height of corrugations were observed as compared to those in the images taken before etching (Figure 4c and Figure 4f). Figure 4i shows the dependence of the corrugation width and height on the etching time, from which the etching rate for the width and height were determined to be 2.6 nm/min and 1.8 nm/min, respectively. The higher etching rate for the width should originate from the developing process. As indicated by the white arrows in Figure 4c, undeveloped material remained at the sides of the land structures. These remaining structures induced DPPs and resulted in a higher etching rate. Table 1 shows the *R*_a_ value that was taken along the nanostripes, from which the decrease in surface roughness along both land structures and grooves is confirmed. In addition, the undeveloped structures at the bottom of the grooves (arrows in Figure 4f and Figure 4h) disappeared. TiO_2_ was transparent to the incident light, so the coherent oscillation of electrons over its periodic structure was negligible. Therefore, plasmonic effects, which can sometimes originate from periodic enhancement of the local field [35], did not contribute to the smoothing of the corrugation pattern. Based on the results in Figure 4, the maximum roughness for which DPP etching will be applicable is in the range of 100 nm if the structure has a small roughness within the roughness envelope (see Figure 1b and Figure 1c), because the land structures of 100 nm in height were etched by using this process.

**Table 1:** Dependence of the surface roughness on the etching time. *A*_L_ is measured along the land structures at position A (Figure 4c and Figure 4d), and *A*_G_ is measured along the grooves at position A (Figure 4c and Figure 4d). *B*_L_ is measured along the land structures at position B (Figure 4f and Figure 4g), and *B*_G_ is measured along the grooves at position B (Figure 4f and Figure 4g).

etching time (min)	*A*_L_ (nm)	*A*_G_ (nm)	*B*_L_ (nm)	*B*_G_ (nm)

0	0.39	0.50	0.41	1.25
10	0.29	0.41	—	—
30	—	—	0.33	0.84

### Dressed photon–phonon desorption

**Figure 5:**
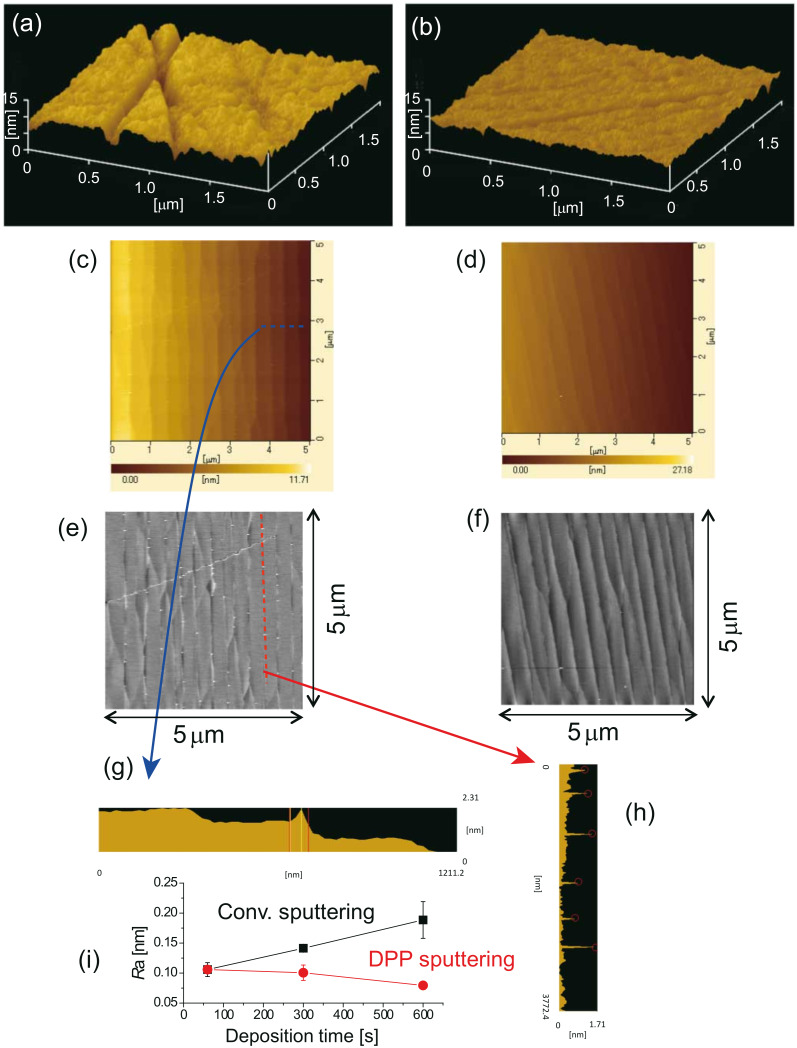
AFM images of the alumina substrate surface after RF sputtering (a) without and (b) with visible light illumination (Reproduced with permission from [46]. Copyright 2010 Springer Science+Business Media). Flattened AFM images of the sapphire substrate surface after RF sputtering (c) without and (d) with visible light illumination. (e, f) Flattened AFM images of (c) and (d), respectively, shown in gray-scale. (g, h) Cross-sectional profiles along the dashed lines in (c) and (e), respectively. (i) Dependence of *R*_a_ on the sputtering time. Solid black squares denote results for conventional RF sputtering, and solid red circles, for RF sputtering under visible light irradiation.

A DPP desorption process has also been developed for smoothing the surfaces of transparent ceramics such as alumina (Al_2_O_3_), which is a hard polycrystalline ceramic [44]. Alumina can be used as a low-loss gain medium for ceramic lasers [45] that are used in laser-driven spark plugs for ignition systems in automobile engines [46]. We expect that the surface roughness (e.g., scratches) of such media could be reduced by sputtering with Al_2_O_3_ nanoparticles, followed by DPP desorption [47]. In this study, radio frequency (RF) sputtering was used to deposit Al_2_O_3_ nanoparticles on an alumina substrate. In the case of conventional RF sputtering, the migration length of the Al_2_O_3_ nanoparticles on the substrate surface depends on the Schwöbel barrier [48] in the free energy profile. The migration length is short near the scratches because the Schwöbel barrier is high at their rims. Thus, the rate of deposition of the Al_2_O_3_ nanoparticles is higher at ridge sites than in flat areas. Hence, since the Al_2_O_3_ nanoparticles preferentially aggregate at the ridges, repairing the scratches by conventional deposition techniques is impossible. To overcome this difficulty, Al_2_O_3_ nanoparticles were deposited under illumination with visible light from a CW laser (λ = 473 nm; power density 2.7 W·cm^−2^) with a wavelength longer than that of the absorption band edge of Al_2_O_3_ (λ_ab_ = 260 nm) [49]. This condition prevented a heating of the substrate surface. Hence, the DPPs generated on the ridges of the scratches activated the Al_2_O_3_ particles and increased their migration length, thereby allowing them to desorb from the ridge [50]. In contrast, the Al_2_O_3_ particles on the slopes and flat regions of the substrate were deposited at the same rate in the absence of DPPs. We note that DPPs were not generated near the bottom of the scratches because the substrate material around this area did not have dimensions at the nanometer-scale, and thus coherent phonons could not be excited. Deposition at the ridges was suppressed by this phonon-assisted process, whereas the bottoms of scratches were filled with Al_2_O_3_ particles. In this way, the scratches were finally repaired. Again, this demonstrates self-organized smoothing of the surface by using DPPs. Figure 5a and Figure 5b show AFM images before and after the RF sputtering of Al_2_O_3_ (30 min sputtering time) under visible light illumination. The average width of the scratches was found to decrease from 128 to 92 nm when this method was adopted, according to a statistical analysis, which employed a Hough transform. Furthermore, the average depth decreased from 3 to 1 nm.

In order to confirm the selective desorption of nanoparticles at the ridge edges, the DPP method was applied to a sapphire substrate with a uniform step-and-terrace structure [51]. Figure 5c and Figure 5d show AFM images after RF sputtering (sputtering time of 30 s), without and with visible light illumination, respectively. To determine the sites of nanoparticle deposition, we obtained the flattened gray-scale images in Figure 5e and Figure 5f, which correspond to Figure 5c and Figure 5d, respectively. Figure 5g and Figure 5h show the cross-sectional profiles along the dashed lines in Figure 5c and Figure 5e, respectively. These images confirm the selective deposition at the terrace edges during conventional RF sputtering. Furthermore, as a further confirmation that DPP desorption prevented the growth of Al_2_O_3_ nanoparticles on the terrace edges, no clear Al_2_O_3_ nanoparticle growth sites were formed during RF sputtering under visible light illumination. After 10 min of RF sputtering under illumination, an ultra-flat sapphire surface with an *R*_a_ value of 0.08 nm was obtained. In contrast, *R*_a_ increased as the sputtering time increased without illumination i.e., when using conventional sputtering (Figure 5i).

## Conclusion

We have reviewed recent progress on the realization of ultraflat materials surfaces. In summary, DPs can be generated in transparent materials when the wavelength used for illumination is longer than the length of the absorption edge of the materials, and the DPP-based technique can be applied to other materials including semiconductors, dielectric materials, insulators, and plastics. DPP etching is a noncontact method and therefore does not cause damage owing to mechanical polishing, and hence, this technique should help to improve the electrical, optical, and/or electro-optical performance of devices in a variety of applications. We also described how the surface roughness changes when the proposed technique is used. Further surface characterization is required to verify that DPP etching is effective for optics and electronics. Because it does not require a contact pad, this technique can also be easily applied for the flattening of larger areas [52] by enlarging the beam spot, or by introducing an LED array. The use of Cl_2_ in the DPP-etching process may induce substrate erosion. Hence, DPP etching requires a vacuum chamber. However, oxygen gas could smooth a diamond substrate at atmospheric pressure. These findings should accelerate the progress of DPP etching of various substrates.
